# Influence of landscape condition on relative abundance and body condition of two generalist freshwater turtle species

**DOI:** 10.1002/ece3.7450

**Published:** 2021-03-24

**Authors:** Joel L. Mota, Donald J. Brown, Danielle M. Canning, Sara M. Crayton, Darien N. Lozon, Alissa L. Gulette, James T. Anderson, Ivana Mali, Brian E. Dickerson, Michael R. J. Forstner, Mark B. Watson, Thomas K. Pauley

**Affiliations:** ^1^ Division of Forestry and Natural Resources West Virginia University Morgantown WV USA; ^2^ Northern Research Station U.S.D.A. Forest Service Parsons WV USA; ^3^ West Virginia Division of Natural Resources Romney WV USA; ^4^ Department of Biology Eastern New Mexico University Portales NM USA; ^5^ Rocky Mountain Research Station U.S.D.A. Forest Service Rapid City SD USA; ^6^ Department of Biology Texas State University San Marcos TX USA; ^7^ Department of Natural Sciences and Mathematics University of Charleston Charleston WV USA; ^8^ Department of Biological Sciences Marshall University Huntington WV USA

**Keywords:** *Chrysemys picta*, habitat generalist, land use, *Trachemys scripta elegans*

## Abstract

Anthropogenic land use changes have broad impacts on biological diversity, often resulting in shifts in community composition. While many studies have documented negative impacts on occurrence and abundance of species, less attention has been given to native species that potentially benefit from anthropogenic land use changes. For many species reaching high densities in human‐dominated landscapes, it is unclear whether these environments represent higher quality habitat than more natural environments. We examined the influence of landscape ecological integrity on relative abundance and body condition of two native generalist freshwater turtle species that are prevalent in anthropogenic systems, the painted turtle (*Chrysemys picta*) and red‐eared slider (*Trachemys scripta elegans*). Relative abundance was negatively associated with ecological integrity for both species, but the relationship was not strongly supported for painted turtles. Body condition was positively associated with ecological integrity for painted turtles, with no strong association for red‐eared sliders. Our study suggests that both species benefitted at the population level from reduced ecological integrity, but individual‐level habitat quality was reduced for painted turtles. The differing responses between these two habitat generalists could partially explain why red‐eared sliders have become a widespread exotic invasive species, while painted turtles have not.

## Graphical abstract

Identifying the differences in the relationship between landscape integrity and body condition for painted turtles and red‐eared sliders may porovide insight on how red‐eared sliders have become a widespread invasive species, while painted turtles have not. We collected and collated turtle capture and morphometric data to create model‐estimated relationships between mean 2.5 km landscape condition value (LCV) and standardized body condition index (BCI) scores for (A) painted turtles (*Chrysemys picta*; *n* = 625) sampled at 46 wetlands across 10 counties in West Virginia and (B) red‐eared sliders (*Trachemys scripta elegans*; *n* = 715) sampled at 42 wetlands across five counties in Texas.

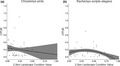

## 1 INTRODUCTION

Anthropogenic land use changes have altered the structure and function of ecosystems on nearly all parts of the planet (Sala et al., 2000; Zwick, 1992). These alterations often reduce the ability of landscapes to support high biological diversity and decrease the systems’ resilience to environmental stressors (i.e., lower their ecological integrity; Freedman, 2015; Ordóñez & Duinker, 2012; Parrish et al., 2003). Much research has been devoted to documenting and quantifying negative impacts of anthropogenic land use changes on wildlife, such as declines in density and local extirpation of populations (Crawford & Bolen, 1976; Wilberg et al., 2011), lower fitness of individuals (Li et al., 2016; Slabbekoorn & Ripmeester, 2008), reduced genetic diversity (Holderegger & Di Giulio, 2010; Miraldo et al., 2016), and behavioral changes (Andersen et al., 2017; Longcore & Rich, 2004). A major outcome of broad‐scale anthropogenic land use change is the loss of habitat specialists and gain of habitat generalist and synanthropic species (including invasive species), with the consequent homogenization of wildlife communities (Clavel et al., 2010; Sofaer et al., 2020).

Habitat generalists often exist at higher densities in human‐dominated landscapes than in more natural landscapes (Fedriani et al., 2001; Roseberry & Woolf, 1998). For example, several studies found that raccoon (*Procyon lotor*) densities in urban and suburban areas were higher than in adjacent rural and undisturbed areas (Prange et al., 2003; Riley et al., 1998). Annual productivity and nesting densities of some bird species, such as Cooper's hawk (*Accipiter cooperi*) and American crow (*Corvus brachyrhynchos*), increase in urban and suburban areas (McGowan, 2001; Rosenfield et al., 1995). Anthropogenic habitat alterations can increase quality of resources such as food and cover, which provides direct benefits to some species (Bateman & Fleming, 2012; West et al., 2016). This is likely the case for strongly synanthropic species, such as raccoon (Demeny et al., 2019; Gross et al., 2012), brown rat (*Rattus norvegicus*; Traweger et al., 2006, Feng & Himsworth, 2014), and house sparrow (*Passer domesticus*; Leu et al., 2008, Khera et al., 2010). Some species can also benefit from changes in thermal conditions associated with anthropogenic land uses (Frishkoff et al., 2015; Leveau, 2018; Miles et al., 2019). For example, Bowne et al., (2018) found the proportion of females in painted turtle (*Chrysemys picta*) populations was positively associated with urbanization, and attributed the finding to higher soil temperatures in urban environments.

While anthropogenic land use changes can increase quality of resources for some species, many generalist species may instead benefit from reduced predation pressure (Eötvös et al., 2018; Rodewald et al., 2011), or reduced interspecific competition for resources. Competitive release occurs when the local distribution or abundance of a species increases in response to decline or extirpation of a resource competitor (Gause, 1932; Hardin, 1960). Many experimental and observational studies have confirmed potential for competitive release (Berger & Gese, 2007; Hairston, 1986; Menge, 1976; Segre et al., 2016). In the context of reduced predation or competition pressure, anthropogenic habitat alterations could both reduce quality of resources and result in increased densities of generalist species (Cruz‐Elizalde et al., 2016; Decena et al., 2020; Peltzer et al., 2006).

In the absence of pre‐ and post‐anthropogenic habitat alteration community data, health of individuals can provide insights into whether generalist species benefit from anthropogenic habitat alterations. Specifically, body condition index (BCI) can be a useful metric to assess habitat quality (Maceda‐Veiga et al., 2014; Pulliam, 2000; Sasaki et al., 2016). A BCI score represents the relationship between the weight and size of an individual relative to the study group, typically using residuals from a log‐transformed length–weight regression (Schulte‐Hostedde et al., 2005). Individuals with BCI scores above the mean have above average amounts of metabolizable tissue (fat or protein) relative to their length and vice versa (Schulte‐Hostedde et al., 2005). Body condition correlates with fitness metrics such as survival probability and fecundity (Bender et al., 2008; Burton et al., 2006; Carranza & Hidalgo de Trucios, 1993).

The painted turtle (*Chrysemys picta*) and red‐eared slider (*Trachemys scripta elegans*) are generalist freshwater turtle species native to North America (Ernst & Lovich, 2009). Our focal subspecies, eastern painted turtle [*C*. *p. picta*] and midland painted turtle [*C*. *p. marginata*], are widely distributed across much of the eastern United States and southeastern Canada (Ernst & Lovich, 2009). The red‐eared slider, a subspecies of the pond slider (*T. scripta*), is native to a large portion of the east‐central United States (Ernst & Lovich, 2009). However, due to their popularity in the pet trade and ability to persist in a wide variety of environmental conditions, non‐native populations of red‐eared sliders have become established in many regions of the world (Héritier et al., 2017; Lambert et al., 2019), and it is considered one of the world's worst invasive species (Lowe et al., 2000). Both species generally prefer shallow lentic freshwater habitats containing a soft mucky bottom with abundant aquatic plants (DonnerWright et al., 1999; Janzen et al., 1992; Morreale & Gibbons, 1986). Both species are also commonly found in wetlands associated with anthropogenic land use, such as agricultural farm ponds and urban retention ponds (Buchanan et al., 2019; Stone et al., 2005). Further, many studies have indicated that densities of painted turtles and red‐eared sliders in anthropogenic wetlands are much higher than other turtle species occupying the same wetlands (Brown, Farallo, et al., 2011; Failey et al., 2007; Glorioso et al., 2010).

Although generalist turtle species can achieve high densities in human‐dominated landscapes, little research has been conducted to assess whether these environments represent higher quality habitat than more natural systems. The purpose of this study was to determine whether relative abundance and body condition of painted turtles sampled in West Virginia, and red‐eared sliders sampled in Texas, are correlated with ecological integrity of the surrounding landscape. We hypothesized that relative abundance of these species would be negatively correlated with ecological integrity, which would suggest that human‐dominated landscapes can support larger populations, potentially due to reduced predation or competition pressure. We also hypothesized that BCI score for these species would be negatively correlated with ecological integrity, which would suggest that habitat quality for these species is better in human‐dominated landscapes.

## 2 METHODS

### 2.1 Species data and sampling sites

We collated turtle capture and measurement data previously collected by the authors for painted turtles in West Virginia and red‐eared sliders in Texas. The data were originally collected for a wide variety of research projects primarily focused on relationships between relative abundance and land use and management (Brown et al., 2012; Gulette, 2018; Mali et al., 2013; Watson & Pauley, 2006) and investigations of hoop net sampling methodology (Gulette et al., 2019; Mali et al., 2014; Oxenrider et al., 2019). For all study sites, turtle populations were sampled using hoop net traps, primarily baited with canned sardines. Turtles were sampled throughout the active season (March–September) in both states. Trap size varied based on study objectives and ranged from 0.3 to 0.91 m diameter in hoop width.

**FIGURE 1:**
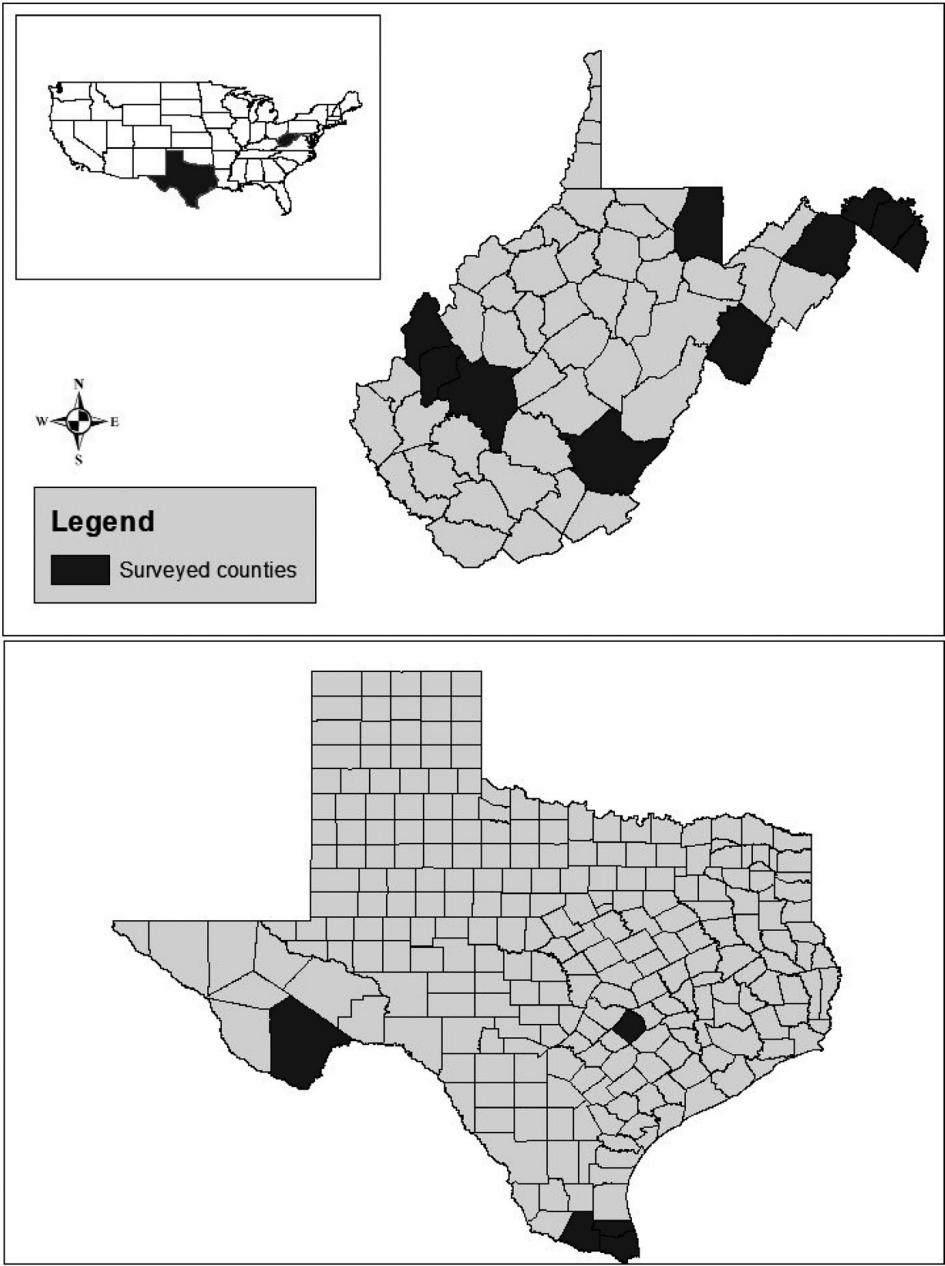
Map of counties sampled for painted turtles (*Chrysemys picta*) in West Virginia (top) and red‐eared sliders (*Trachemys scripta elegans*) in Texas (bottom), USA (inset). We sampled 49 wetlands in West Virginia between 1999 and 2019, and 43 wetlands in Texas between 2008 and 2013

Trapping occurred between 1999 and 2019 in West Virginia, and between 2008 and 2013 in Texas (Appendix S1). Painted turtles were sampled at 49 wetlands across 10 counties in southern and eastern West Virginia (Figure 1; Appendix S1). Red‐eared sliders were sampled at 43 wetlands across five counties in south, central, and west Texas (Figure 1; Appendix S1). Midline carapace length (MCL) was measured to the nearest 1 mm using tree calipers (method D in Iverson & Lewis, 2018). Weight was measured using spring scales to the nearest 1, 5, 10, and 50 g for turtles weighing ≤10, ≤600, ≤2,500, and >2,500 g, respectively (Brown et al., 2020). Turtles were individually marked using marginal scute notches (Cagle, 1939). In both states, sampled wetlands occurred in agricultural systems, river backwaters, and natural areas. In Texas, several wetlands also occurred in heavily urban environments. Wetlands ranged in size from 0.008 to 5.577 ha (median = 0.063 ha) in West Virginia and 0.018 to 66.264 ha (median = 1.145 ha) in Texas.

### 2.2 Landscape condition

We used NatureServe's Landscape Condition Model (LCM) for Temperate North America as our landscape condition index (Hak & Comer, 2017). This index is based on 20 landscape characteristics, categorized as transportation (including roads at multiple classification levels), urban and industrial development, and managed and modified land cover (Hak & Comer, 2017). Ecological integrity is scored from 0 to 1, with scores close to 0 and 1 representing areas of lowest and highest ecological integrity, respectively (Hak & Comer, 2017). The model has a spatial resolution of 90 m^2^. We digitized sampled wetland boundaries using aerial imagery and created buffers around each water body at 0.1 km, 1.0 km, and 2.5 km. The 0.1, 1.0, and 2.5 km buffers were specified to approximate local wetland, home range size, and dispersal area size buffers for our focal turtle species (Bodie & Semlitsch, 2000; Bowne & White, 2004; Gibbons, 1967; Tucker & Lamer, 2008). We computed the mean landscape condition value within each buffer.

The LCM provides a static measure of ecological integrity and was constructed using datasets representing environmental conditions between approximately 2003 and 2010, including the 2011 National Landcover Database (NLCD; Hak & Comer, 2017). We performed a preliminary analysis to ensure the land cover surrounding our sites was similar between the turtle sampling and LCM timeframes. We obtained the NLCD data for the years 2001, 2006, 2011, and 2016, clipped the layers to a 2.5 km buffer around each site, and computed Pearson's correlation coefficients between the 2011 NLCD layer and the layer that most closely matched the turtle sampling timeframe. Pearson's correlation coefficients were ≥0.9588 across all sites, indicating the LCM likely provides a reliable metric of ecological integrity for our sites. All spatial analyses were performed using ArcMAP 10.6 (ESRI, Redlands, California, USA).

### 2.3 Turtle relative abundance

Trapping effort varied among wetlands, and thus, we used captures‐per‐unit‐effort (CPUE) as our metric of relative abundance (Brown et al., 2011; Murray & Seed, 2010). To obtain CPUE, we divided the number of unique individual captures of the focal species by the number of trap days (TD) at each wetland, where a single TD represented one trap in the water for one day (Appendix S1). To minimize potential CPUE biases due to level of trapping effort, we specified a target effort of 50 TD and removed sites with <20 TD (*n* = 2). For sites with >50 TD, we excluded all subsequent days of trapping once the site reached 50 TD. For the analysis, site‐level TD ranged from 20 to 123 (mean = 54, hereafter reduced CPUE analysis; Appendix S1). We supplemented the reduced CPUE analysis with an additional analysis that included all TD at each site (hereafter full CPUE analysis; Appendices S2 and S3). For both analyses, we excluded 9 sites in West Virginia because number of trap days was not available.

### 2.4 Turtle body condition

We computed turtle BCI scores using the residuals of log‐transformed MCL‐weight regressions (Schulte‐Hostedde et al., 2005). We computed BCIs separately for each sex within each species and standardized the values (0 mean, 1 standard deviation) so that BCI scores were weighted equally among each sex and species (Schulte‐Hostedde et al., 2005). We removed adult and subadult turtle capture records with unrecorded sex from the dataset. We also excluded juveniles, including painted turtles <89 mm MCL (Balcombe & Licht, 1987; Lefevre & Brooks, 1995) and red‐eared sliders <101 mm MCL (Cagle, 1948) because sex was unknown for this size class.

### 2.5 Statistical analyses

We used linear mixed‐effects models to analyze the relationship between landscape condition and turtle CPUE and BCI (Zuur et al., 2009). For CPUE, we grouped wetlands into four size classes (Class 1 = <2.750 ha; Class 2 = 2.750 ha < 10.795 ha; Class 3 = 10.795 ha < 33.615 ha; Class 4 = ≥35.615 ha) using the Jenks natural breaks classification method (Jenks, 1977). This method is based on Fisher's “Exact Optimization” method (Fischer, 1958), which seeks to optimize homogeneity within groups by minimizing the sum of squares difference. We included size class as a random effect to account for potential effects of wetland size on CPUE. Fixed effects included landscape condition value (LCV) and trap size. For this analysis, we used the LCV extent that was most supported for the BCI analysis. We specified traps as small (0.3 m) or large (0.76–0.91 m) to account for potential trap size effects on CPUE of painted turtles in West Virginia. Trap size (0.76 m) was consistent for all wetlands sampled for red‐eared sliders in Texas. For CPUE, preliminary analyses indicated the LCV relationship may be quadratic, and thus we tested LCV as both a linear and quadratic predictor. For BCI, we included wetland as a random effect to account for site‐level environmental variation independent of landscape condition that could influence BCI. Fixed effects included sex and mean LCV surrounding the wetland at distances of 0.1, 1.0, and 2.5 km. We tested the influence of sex as both an additive effect and an interactive effect.

We used Akaike's information criterion corrected for small sample size (AIC_c_) to rank candidate models (Burnham & Anderson, 2004). We considered models to have strong support if ∆AICc < 2 (Burnham et al., 2011). For the most supported models, we assessed confidence for an effect of each variable by computing the 85% confidence intervals (CI) of the beta coefficients (Arnold, 2010) and considered there to be evidence for a strong effect when CIs did not overlap zero (Halsey, 2019). For all analyses, we assessed assumptions of normality using quantile–quantile plots and homoscedasticity using residual plots (Zuur et al., 2009, 2010). For the CPUE models, we removed one painted turtle site to satisfy the assumption of normality. For the BCI models, we removed 23 extreme outliers (>4 standard deviations from the mean), which likely represented incorrect MCL or weight measurements. All analyses were conducted using program R (version 3.6.3). We performed the Jenks natural breaks classification using the package BAMMtools (version 2.1.7) and assessed model assumptions using the package car (version 3.0‐6). We created mixed‐effects models using the package nlme (version 3.1‐142), performed model selection analyses using the package AICcmodavg (version 2.2‐2), and plotted results using the package ggplot2 (version 3.2.1).

## 3 RESULTS

For the reduced CPUE dataset, CPUE per wetland ranged from 0.02 to 1.00 (mean = 0.22) for painted turtles in West Virginia and from 0 to 0.70 (mean = 0.14) for red‐eared sliders in Texas (Appendix S1). For the BCI dataset, unique turtle captures per wetland ranged from 1 to 109 (mean = 14) for painted turtles in West Virginia and from 1 to 135 (mean = 17) for red‐eared sliders in Texas (Appendix S1). The LCV scores ranged from 0.016 to 0.6 (mean = 0.221) for sampled wetlands in West Virginia and from 0.005 to 0.890 (mean = 0.437) for sampled wetlands in Texas. The LCV scores were highly correlated among the three buffer sizes within each state (*r* ^2^ = .82–.96), indicating landscape condition near the wetland was similar to landscape condition in the surrounding landscape, at least at the spatial resolution of the LCM.

**FIGURE 2:**
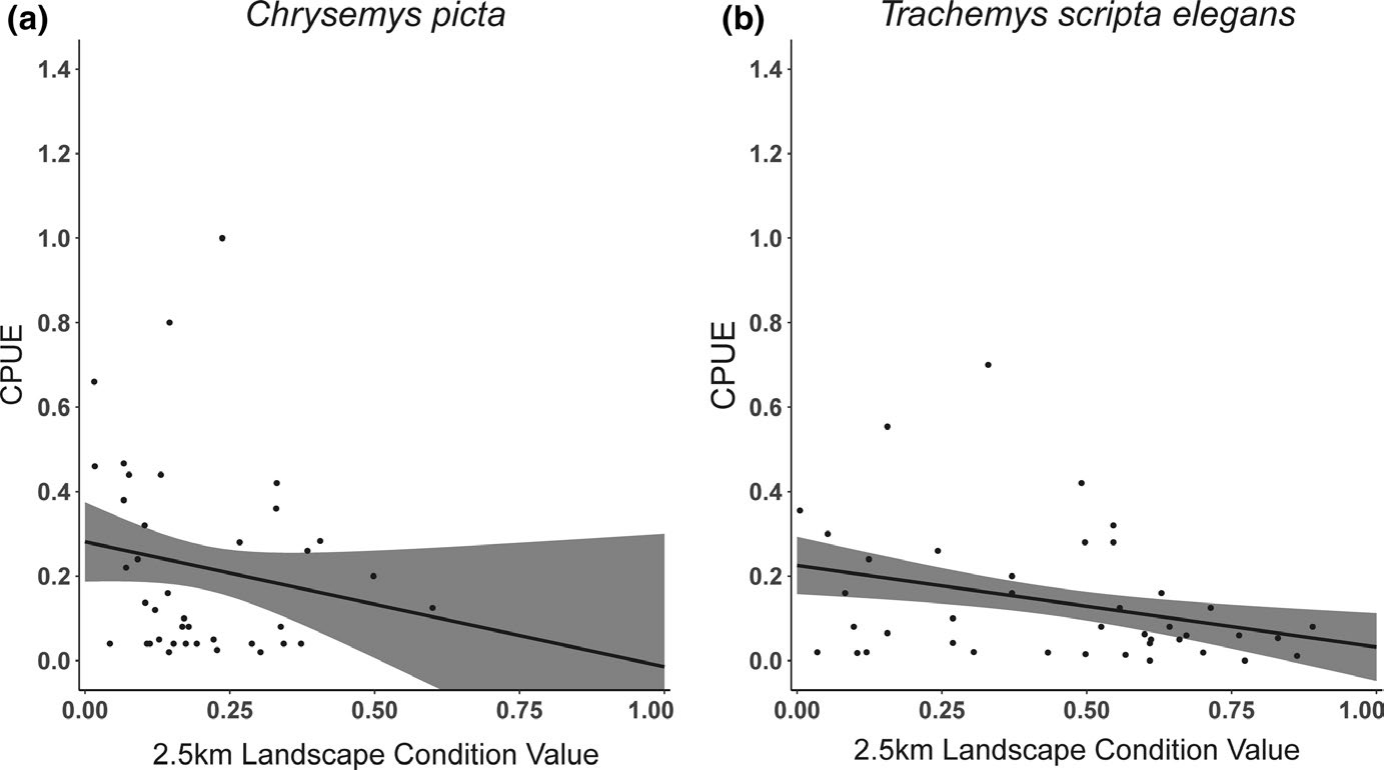
Model‐estimated relationship between mean 2.5 km landscape condition value (LCV) and captures‐per‐unit‐effort (CPUE) for (a) 39 painted turtle (*Chrysemys picta*) wetlands located across 10 counties in West Virginia, and (b) 41 red‐eared slider (*Trachemys scripta elegans*) wetlands located across five counties in Texas using the reduced CPUE analysis dataset. Wetlands where trap days could not be calculated were excluded from this analysis. We included wetland size as a random effect in analyses to account for the influence of size on CPUE. Black circles depict observed CPUE, and gray bands depict 85% confidence intervals. Note the maximum LCV for *C. picta* sites was 0.6

**FIGURE 3:**
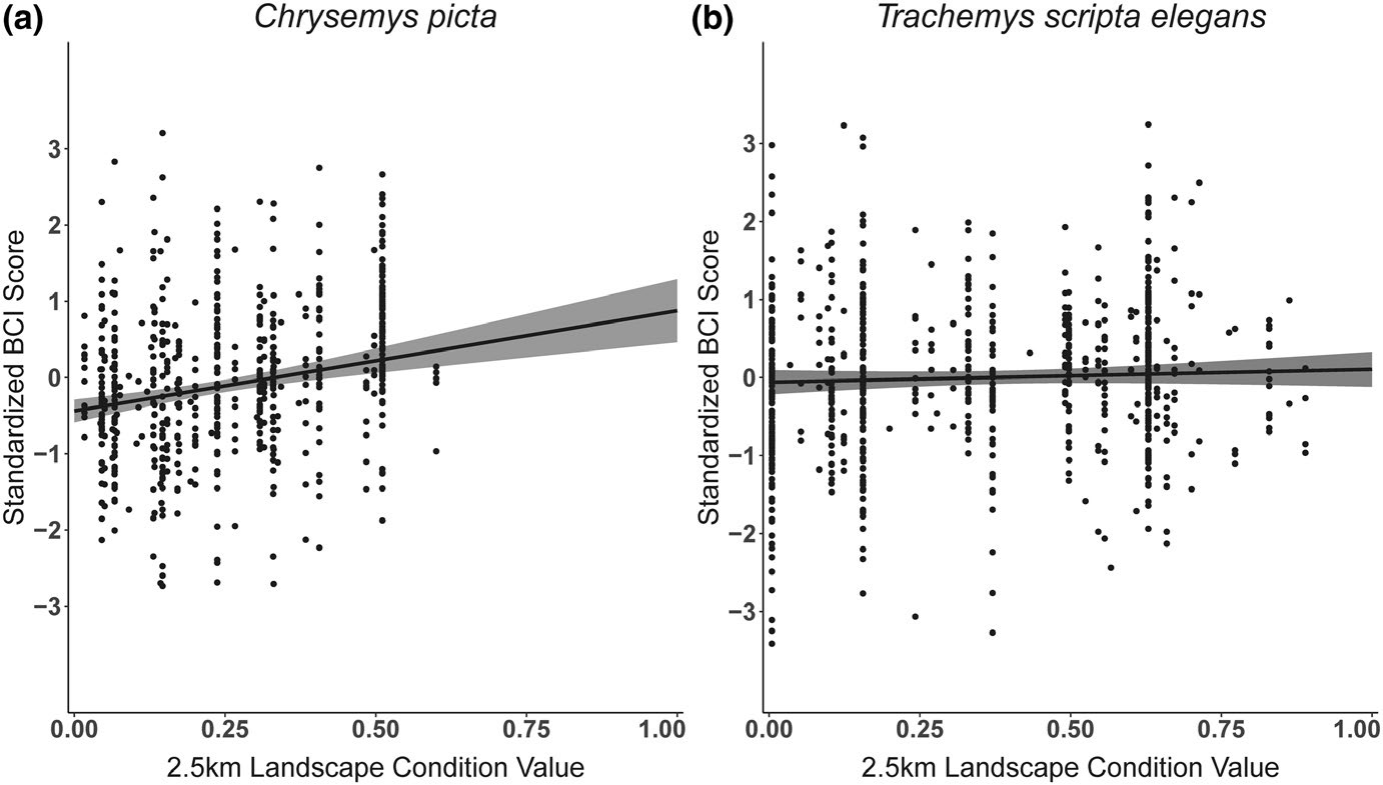
Model‐estimated relationships between mean 2.5 km landscape condition value (LCV) and standardized body condition index (BCI) scores for (a) painted turtles (*Chrysemys picta*; *n* = 625) sampled at 46 wetlands across 10 counties in West Virginia and (b) red‐eared sliders (*Trachemys scripta elegans*; *n* = 715) sampled at 42 wetlands across 5 counties in Texas. We included wetland as a random effect in analyses to account for site‐level environmental variation independent of landscape condition that could influence BCI. Black circles depict standardized BCI values, and gray bands depict 85% confidence intervals. Note the maximum LCV for *C. picta* sites was 0.6

For the painted turtle reduced CPUE analysis, the most supported model was the null model (*w_i_* = 0.51; Table 1). The second most supported model was the linear 2.5 km LCV model (*w_i_* = 0.26, ΔAIC_c_ = 1.31). For this model, predicted CPUE decreased by 0.296 as LCV increased from 0 to 1 (Figure 2a), but the CI broadly overlapped zero (−0.695–0.104). We obtained similar results for the full CPUE analysis (Appendices S2 and S3). For painted turtle BCI, the linear 2.5 km LCV model was the most supported model (*w_i_* = 0.28; Table 1). The linear 2.5 km LCV + sex (*w_i_* = 0.18, ΔAIC_c_ = 0.90) and linear 1.0 km LCV (*w_i_* = 0.13, ΔAIC_c_ = 1.51) models also had strong support. The null model received the lowest support (*w_i_* = 0.01; Table 1). For the most supported model, predicted BCI increased 1.32 standard deviations as LCV increases from 0–1 (Figure 3a), and the CI did not overlap zero (0.782–1.849).

**TABLE 1:** Model selection results for the influence of landscape integrity (landscape condition value [LCV]) on captures‐per‐unit‐effort (CPUE) and body condition index (BCI) of painted turtles (*Chrysemys picta*) in West Virginia and red‐eared sliders (*Trachemys scripta elegans*) in Texas

Model	AIC_c_	ΔAIC_c_	*w_i_*
***Chrysemys picta***			
*CPUE*			
(.)	2.08	0.00	0.51
LCV_2.5_	3.39	1.31	0.26
LCV_2.5_ (Q)	5.08	3.00	0.11
LCV_2.5_ + Trap size	5.82	3.74	0.08
LCV_2.5_ (Q) + Trap size	7.56	5.48	0.03
*BCI*			
LCV_2.5_	1,707.23	0.00	0.28
LCV_2.5_ + Sex	1,708.13	0.90	0.18
LCV_1.0_	1,708.74	1.51	0.13
LCV_0.1_	1,708.78	1.56	0.13
LCV_2.5_ × Sex	1,709.46	2.23	0.09
LCV_1.__0_ + Sex	1,709.99	2.76	0.07
LCV_0.1_ + Sex	1,710.21	2.98	0.06
LCV_1.__0_ × Sex	1,711.51	4.28	0.03
LCV_0.1_ × Sex	1,712.12	4.89	0.02
(.)	1,714.15	6.93	0.01
***Trachemys scripta elegans***			
*CPUE*			
LCV_2.5_	−31.25	0.00	0.53
LCV_2.5_ (Q)	−29.75	1.49	0.25
(.)	−29.48	1.76	0.22
*BCI*			
(.)	2,011.47	0.00	0.36
LCV_2.5_	2,013.08	1.61	0.16
LCV_1.0_	2,013.47	1.99	0.13
LCV_0.1_	2,013.49	2.02	0.13
LCV_2.5_ + Sex	2,015.10	3.63	0.06
LCV_1.__0_ + Sex	2,015.49	4.01	0.05
LCV_0.1_ + Sex	2,015.52	4.04	0.05
LCV_2.5_ × Sex	2,017.13	5.66	0.02
LCV_1.__0_ × Sex	2,017.49	6.02	0.02
LCV_0.1_ × Sex	2,017.54	6.07	0.02

Note

For CPUE, we used a reduced trapping dataset with a target of 50 trap days per site. We used Akaike's information criterion corrected for small sample size (AIC_c_) to rank candidate models. For CPUE, we used the 2.5 km LCV and tested a linear and quadratic (*Q*) relationship. The size of traps (Trap Size) varied at West Virginia sites and was included as a candidate predictor for *C. picta*. For BCI, we ranked mean LCV at 0.1, 1.0, and 2.5 km surrounding wetlands. We also tested the influence of sex as an additive and interactive predictor at the three spatial scales. We standardized BCI by species and sex prior to analysis. The null model is shown as (.) and includes only the intercept. Wetland buffer distance is denoted by subscripts following the LCV term. Akaike weights are represented as *w_i_*.

For the red‐eared slider reduced CPUE analysis, the most supported model was the linear 2.5 km LCV model (*w_i_* = 0.53), but the quadratic 2.5 km LCV (*w_i_* = 0.25, ΔAIC_c_ = 1.49) and null (*w_i_* = 0.22, ΔAIC_c_ = 1.76) models also had strong support (Table 1). The linear 2.5 km LCV model‐predicted CPUE decreased 0.19 standard deviations as LCV increased from 0 to 1, and 85% CI did not overlap zero (−0.058 to −0.328; Figure 2b). The modeled relationship was similar for the full CPUE analysis (Appendix S3), except the quadratic 2.5 km LCV model received higher support than the linear model (Appendix S2). For red‐eared slider BCI, the most supported model was the null model (*w_i_* = 0.36; Table 1). The linear 2.5 km LCV (*w_i_* = 0.16, ΔAIC_c_ = 1.61) and linear 1.0 km LCV (*w_i_* = 0.13, ΔAIC_c_ = 1.99) models also had strong support. For the linear 2.5 km LCV model, predicted BCI increased 0.16 standard deviations as LCV increases from 0 to 1 (Figure 3b). However, the 85% CI broadly overlapped zero (−0.174–0.503).

## 4 DISCUSSION

Generalist species often appear to benefit from anthropogenically degraded landscapes, but the underlying causes that enable increased densities of most habitat generalists are not clear. We sought to improve our understanding of the underlying forces allowing two generalist turtle species to maintain high abundances in highly degraded systems. Our results provide some support for the hypothesis that generalist freshwater turtle species benefit from anthropogenic land use, as relative abundance of both species was negatively associated with ecological integrity. However, the two species differed in individual‐level responses, with reduced ecological integrity appearing to negatively impact painted turtles but not red‐eared sliders. This indicates that painted turtles may benefit from anthropogenic land uses through other factors than improved habitat quality, such as reduced predation or competition pressure, which has been documented for other freshwater turtle species (Petrozzi et al., 2021; Ryan et al., 2008; Spencer & Thompson, 2005).

It is interesting that individual‐level responses to ecological integrity differed between these two generalist turtle species, as both species are prevalent in wetlands that span a wide range of environmental conditions (Brown et al., 2012; Buchanan et al., 2019). Wetlands associated with anthropogenic landscapes generally differ from those in more natural systems. For example, wetlands associated with developed and working lands are often more eutrophic (Kennish, 2002; Smith & Schindler, 2009), which in turn influences many abiotic and biotic factors (McCormick & Laing, 2003; McGoff et al., 2013; Naselli‐Flores & Barone, 2000). Created wetlands (e.g., farm ponds and mitigation wetlands) also tend to be deeper than natural wetlands (Cole & Brooks, 2000; Cole et al., 2006; Gamble & Mitsch, 2009). These wetland‐specific characteristics likely interact with the surrounding landscape condition to influence habitat quality for the two species (Buchanan et al., 2019; Cosentino et al., 2010; Ryan et al., 2008). While our study was not designed to control for wetland characteristics, we encourage future studies to explore interactions between landscape integrity and species‐specific wetland habitat quality.

The red‐eared slider has successfully established non‐native populations in many regions of the world (e.g., France [García‐Díaz et al., 2017], Japan [Kakuda et al., 2019], South Korea [Oh et al., 2017]). Our results suggest that even within their native distribution (apart from one study site in west Texas), red‐eared sliders benefit from environmental conditions associated with lower ecological integrity. The ability to exploit anthropogenic habitats, in conjunction with potentially reduced competitive pressure in anthropogenically altered systems (Cadi & Joly, 2003), could explain why red‐eared sliders are a particularly successful invasive species. In contrast, painted turtles did not appear to strongly benefit from lower ecological integrity and are also not a prominent invasive species, despite also being common in the pet trade (Hohn, 2003; Telecky, 2001). Interestingly, red‐eared sliders typically achieve much higher densities than painted turtles in sympatric areas (Bodie et al., 2000; Dreslik et al., 2005), indicating red‐eared sliders may be competitively dominant (Lindeman, 1999; Polo‐Cavia et al., 2011).

Anthropogenic land use changes result in creation, loss, and alteration of environmental conditions, resulting in wildlife species “winners and losers” (McKinney & Lockwood, 1999). Globally, freshwater turtles are declining in human‐dominated systems due to a variety of pressures, such as habitat loss and degradation, and overexploitation for food or pets (Gibbons et al., 2000; Lovich et al., 2018). Further, the general life history strategy of freshwater turtles is characterized by a long lifespan, delayed sexual maturity, and low annual recruitment (Congdon et al., 1994), which can result in both slow declines and slow recovery rates (e.g., Howell et al., 2019; Mullin et al., 2020). Our investigation of the relationship between landscape integrity and habitat quality for two widely distributed habitat generalist turtles in North America suggests that ecological integrity has little influence on habitat quality for the red‐eared slider, potentially explaining its prominence as an exotic invasive species (Lowe et al., 2000), and ecological degradation could benefit both species at the population level. Thus, as many regions in North America continue to shift toward heavy anthropogenic use (e.g., agriculture and urbanization; Brown et al., 2005; Ordonez et al., 2014), we expect these two species to be “winners” in comparison with other sympatric freshwater turtle species.

## CONFLICT OF INTEREST

The authors declare no competing interests or conflicts of interest.

## AUTHOR CONTRIBUTION

**Joel L. Mota:** Conceptualization (equal); Data curation (lead); Formal analysis (lead); Investigation (lead); Methodology (lead); Supervision (supporting); Visualization (lead); Writing‐original draft (lead); Writing‐review & editing (equal). **Donald J. Brown:** Conceptualization (equal); Data curation (supporting); Formal analysis (supporting); Funding acquisition (equal); Investigation (equal); Methodology (supporting); Project administration (lead); Resources (lead); Software (lead); Supervision (lead); Validation (lead); Visualization (supporting); Writing‐original draft (supporting); Writing‐review & editing (equal). **Danielle M. Canning:** Conceptualization (equal); Data curation (supporting); Formal analysis (supporting); Investigation (supporting); Methodology (supporting); Writing‐original draft (supporting); Writing‐review & editing (supporting). **Sara M. Crayton:** Formal analysis (supporting); Investigation (supporting); Writing‐original draft (supporting); Writing‐review & editing (supporting). **Darien N. Lozon:** Data curation (supporting); Writing‐review & editing (supporting). **Alissa L. Gulette:** Data curation (supporting); Writing‐review & editing (supporting). **James T. Anderson:** Funding acquisition (equal); Writing‐review & editing (supporting). **Ivana Mali:** Data curation (supporting); Writing‐review & editing (supporting). **Brian E. Dickerson:** Data curation (supporting); Writing‐review & editing (supporting). **Michael R. J. Forstner:** Data curation (supporting); Funding acquisition (equal); Writing‐review & editing (supporting). **Mark B. Watson:** Data curation (supporting); Funding acquisition (equal); Writing‐review & editing (supporting). **Thomas K. Pauley:** Data curation (supporting); Funding acquisition (equal); Writing‐review & editing (supporting).

## Supporting information

Appendix S1‐S3

## Data Availability

The data used in this study are achieved in the Dryad data repository: https://doi.org/10.5061/dryad.jdfn2z39x.
