# Supplementary material for: Influence of landscape condition on relative abundance and body condition of two generalist freshwater turtle species
Source: Ecol Evol. 2021 Mar 24;11(10):5511–21. doi: 10.1002/ece3.7450 (PMC8131803; doi:10.1002/ece3.7450)
Supplement: Supplementary file 1 — Appendix S1‐S3 [file ECE3-11-5511-s001.docx]

Appendix 1. Wetland-level information for red-eared slider (*Trachemys scripta elegans*) sites in Texas (n = 43) and painted turtle (*Chrysemys picta*) sites in West Virginia (n = 50), including site names (Site), state (Texas [TX] or West Virginia [WV]), county, years the wetland was sampled (Years), size of the site (area of wetland; ha), landscape condition value (LCV; 2.5 km buffer), number of turtles included in the body condition analysis (n [BCI]), number of unique individuals captured for the reduced captures-per-unit-effort (CPUE) analysis (n [Reduced]), number of trap days for the reduced CPUE analysis (Trap Days [Reduced]), CPUE for the reduced CPUE analysis (CPUE [Reduced]), number of unique individuals captured for the full CPUE analysis (n [Full]), total number of trap days (Trap Days [Full]), and CPUE for the full CPUE analysis (CPUE [Full]). Two sites were removed for the reduced CPUE analysis due to low trapping effort, and 10 sites were removed from both the reduced CPUE and full CPUE analyses due to missing data on trapping effort, which are shown in the table as NA.

| Site | State | County | Years | Size (ha) | LCV | n (BCI) | n (Reduced) | Trap Days (Reduced) | CPUE (Reduced) | n (Full) | Trap Days (Full) | CPUE (Full) |
| --- | --- | --- | --- | --- | --- | --- | --- | --- | --- | --- | --- | --- |
| GLR 1 | TX | Bastrop | 2010-2012 | 0.089 | 0.643 | 10 | 4 | 50 | 0.085 | 10 | 118 | 0.085 |
| GLR 10 | TX | Bastrop | 2010 | 0.018 | 0.609 | 2 | 2 | 49 | 0.041 | 2 | 89 | 0.022 |
| GLR 11 | TX | Bastrop | 2009-2013 | 0.16 | 0.609 | 4 | 0 | 49 | 0 | 6 | 131 | 0.046 |
| GLR 12 | TX | Bastrop | 2009-2013 | 0.281 | 0.672 | 11 | 3 | 50 | 0.06 | 11 | 270 | 0.041 |
| GLR 13 | TX | Bastrop | 2012 | 0.031 | 0.611 | 1 | 1 | 20 | 0.05 | 1 | 20 | 0.05 |
| GLR 15 | TX | Bastrop | 2010-2012 | 0.029 | 0.433 | 1 | 1 | 52 | 0.019 | 1 | 92 | 0.011 |
| GLR 16 | TX | Bastrop | 2009-2013 | 0.197 | 0.305 | 5 | 1 | 49 | 0.0204 | 5 | 249 | 0.020 |
| GLR 2 | TX | Bastrop | 2009-2013 | 0.069 | 0.701 | 6 | 1 | 52 | 0.019 | 6 | 242 | 0.025 |
| GLR 5 | TX | Bastrop | 2012 | 0.031 | 0.714 | 4 | 4 | 32 | 0.125 | 4 | 32 | 0.125 |
| GLR 9 | TX | Bastrop | 2010-2013 | 0.053 | 0.66 | 11 | 4 | 80 | 0.075 | 15 | 200 | 0.075 |
| Welsh Pond | TX | Bastrop | 2010 | 0.082 | 0.6 | 5 | 5 | 80 | 0.063 | 5 | 80 | 0.063 |
| Beaver Pond BBNP | TX | Brewster | 2010 | 0.326 | 0.863 | 2 | 1 | 90 | 0.011 | 3 | 360 | 0.008 |
| Heron Cove | TX | Cameron | 2009 | 0.336 | 0.525 | 7 | 4 | 50 | 0.08 | 7 | 100 | 0.07 |
| Lake FM 511 | TX | Cameron | 2009 | 9.422 | 0.546 | 13 | 14 | 50 | 0.28 | 14 | 50 | 0.28 |
| Laredo Street | TX | Cameron | 2009 | 0.217 | 0.491 | 19 | 21 | 50 | 0.42 | 21 | 50 | 0.42 |
| Los Ebanos Preserve | TX | Cameron | 2009 | 0.965 | 0.269 | 5 | 5 | 50 | 0.1 | 5 | 90 | 0.056 |
| Pond CR 1577 | TX | Cameron | 2009-2011 | 4.501 | 0.497 | 36 | 14 | 50 | 0.247 | 37 | 150 | 0.0247 |
| Resaca California Rd | TX | Cameron | 2010-2013 | 4.429 | 0.557 | 19 | 10 | 80 | 0.125 | 20 | 231 | 0.087 |
| Resaca de la Palma SP | TX | Cameron | 2009 | 3.432 | 0.89 | 4 | 4 | 50 | 0.08 | 4 | 90 | 0.044 |
| Resaca Timothy Rd | TX | Cameron | 2009 | 26.998 | 0.053 | 8 | 8 | 50 | 0.16 | 8 | 50 | 0.16 |
| Resaca Southmost Preserve | TX | Cameron | 2008-2013 | 3.833 | 0.629 | 135 | 15 | 50 | 0.3 | 164 | 1625 | 0.101 |
| Reservoir FM 1595 | TX | Cameron | 2008-2009 | 7.601 | 0.156 | 8 | 8 | 123 | 0.065 | 8 | 123 | 0.065 |
| Reservoir FM 508 | TX | Cameron | 2009 | 27.983 | 0.1998 | 1 | NA | NA | NA | 1 | 5 | 0.2 |
| Reservoir Los Indios City | TX | Cameron | 2009 | 66.264 | 0.763 | 1 | 3 | 50 | 0.02 | 3 | 50 | 0.06 |
| Bass Lake | TX | Hidalgo | 2009-2013 | 0.468 | 0.371 | 22 | 8 | 50 | 0.16 | 22 | 230 | 0.096 |
| Bentsen-Rio Grande SP | TX | Hidalgo | 2008 | 8.306 | 0.567 | 1 | 1 | 73 | 0.014 | 1 | 212 | 0.005 |
| Edinburg Scenic Wetlands | TX | Hidalgo | 2008-2013 | 4.332 | 0.005 | 129 | 27 | 76 | 0.36 | 139 | 1076 | 0.129 |
| Estero Llano Grande SP Grebe Marsh | TX | Hidalgo | 2009-2013 | 0.235 | 0.498 | 1 | 1 | 55 | 0.018 | 1 | 70 | 0.014 |
| Estero Llano Grande SP Ibis Pond | TX | Hidalgo | 2009-2011 | 2.083 | 0.371 | 23 | 14 | 70 | 0.2 | 24 | 185 | 0.130 |
| Frontera Audubon | TX | Hidalgo | 2008-2013 | 0.171 | 0.104 | 24 | 1 | 55 | 0.018 | 30 | 250 | 0.12 |
| Reservoir Jasmin Road | TX | Hidalgo | 2009 | 35.615 | 0.035 | 1 | 1 | 50 | 0.02 | 1 | 50 | 0.02 |
| Reservoir Old Hidalgo | TX | Hidalgo | 2009 | 2.304 | 0.269 | 2 | 2 | 48 | 0.042 | 2 | 73 | 0.027 |
| Santa Ana NWR Cattail Lake | TX | Hidalgo | 2008-2011 | 10.795 | 0.773 | 4 | 0 | 55 | 0 | 4 | 265 | 0.015 |
| Santa Ana NWR Pintail Lake | TX | Hidalgo | 2008-2011 | 3.85 | 0.83 | 13 | 4 | 75 | 0.053 | 13 | 541 | 0.024 |
| Unnamed Street | TX | Hidalgo | 2009 | 2.546 | 0.098 | 5 | 4 | 50 | 0.08 | 5 | 86 | 0.058 |
| Pond 15276 | TX | Willacy | 2008-2013 | 2.134 | 0.156 | 91 | 36 | 65 | 0.553 | 103 | 255 | 0.404 |
| Pond BUS 77 | TX | Willacy | 2009 | 0.101 | 0.083 | 8 | 8 | 50 | 0.16 | 8 | 50 | 0.16 |
| Pond CR 375 | TX | Willacy | 2009 | 0.131 | 0.278 | 1 | NA | NA | NA | 2 | 5 | 0.4 |
| Pond CR 465 | TX | Willacy | 2009 | 0.218 | 0.33 | 34 | 35 | 50 | 0.7 | 35 | 50 | 0.7 |
| Pond FM 2099 | TX | Willacy | 2009 | 2.75 | 0.243 | 12 | 13 | 50 | 0.26 | 13 | 50 | 0.26 |
| Pond HWY 77 | TX | Willacy | 2008-2009 | 3.374 | 0.546 | 16 | 16 | 50 | 0.32 | 16 | 50 | 0.32 |
| Reservoir Bulldog | TX | Willacy | 2009 | 1.324 | 0.124 | 10 | 12 | 50 | 0.24 | 12 | 50 | 0.24 |
| Reservoir Palo Alto | TX | Willacy | 2009 | 0.247 | 0.12 | 0 | 1 | 50 | 0.02 | 1 | 50 | 0.02 |
| BAA | WV | Berkeley | 2017-2019 | 0.126 | 0.237 | 64 | 50 | 50 | 1 | 77 | 70 | 1.1 |
| BAAR | WV | Berkeley | 2017 | 0.015 | 0.384 | 12 | 13 | 50 | 0.26 | 13 | 50 | 0.26 |
| GBA | WV | Greenbriar | 2017 | 0.019 | 0.511 | 109 | 114 | 50 | 2.28* | 114 | 50 | 2.28 |
| GBAR | WV | Greenbriar | 2017 | 0.008 | 0.498 | 7 | 10 | 50 | 0.2 | 10 | 50 | 0.2 |
| Capon River Rd 2 | WV | Hampshire | 2019 | 0.095 | 0.33 | 32 | 18 | 50 | 0.36 | 48 | 120 | 0.4 |
| Edwards Run WMA | WV | Hampshire | 2019 | 0.707 | 0.406 | 36 | 17 | 60 | 0.283 | 44 | 180 | 0.244 |
| 51 Turkey Run | WV | Jefferson | 2019 | 0.177 | 0.128 | 1 | 1 | 20 | 0.05 | 1 | 20 | 0.05 |
| Altona Marsh | WV | Jefferson | 2019 | 0.455 | 0.067 | 45 | 28 | 60 | 0.467 | 50 | 180 | 0.278 |
| Cool springs | WV | Jefferson | 2019 | 0.081 | 0.222 | 0 | 1 | 20 | 0.05 | 1 | 20 | 0.05 |
| Harewood | WV | Jefferson | 2019 | 0.148 | 0.171 | 9 | 5 | 50 | 0.1 | 22 | 120 | 0.183 |
| Leetown | WV | Jefferson | 2019 | 0.067 | 0.104 | 14 | 7 | 51 | 0.137 | 21 | 204 | 0.103 |
| Armour Creek | WV | Kanawha | 1999-2000 | 0.608 | 0.05 | 10 | 10 | NA | NA | 10 | NA | NA |
| Bills Creek | WV | Kanawha | 1999-2000 | 0.218 | 0.133 | 12 | 12 | NA | NA | 12 | NA | NA |
| Crooked Creek | WV | Kanawha | 1999-2000 | 0.198 | 0.046 | 41 | 44 | NA | NA | 44 | NA | NA |
| Guano Creek | WV | Kanawha | 1999 | 0.174 | 0.315 | 11 | 11 | NA | NA | 11 | NA | NA |
| Manila Creek | WV | Kanawha | 1999 | 0.65 | 0.308 | 46 | 48 | NA | NA | 48 | NA | NA |
| Nine Mile Creek | WV | Mason | 1999 | 0.62 | 0.174 | 14 | 16 | NA | NA | 16 | NA | NA |
| MMA | WV | Mason | 2016 | 0.022 | 0.338 | 4 | 4 | 50 | 0.08 | 4 | 50 | 0.08 |
| MMAR | WV | Mason | 2016 | 0.018 | 0.267 | 12 | 14 | 50 | 0.28 | 14 | 50 | 0.28 |
| MMBR | WV | Mason | 2016 | 0.012 | 0.145 | 1 | 1 | 50 | 0.02 | 1 | 50 | 0.02 |
| MMC | WV | Mason | 2016 | 0.017 | 0.303 | 1 | 1 | 50 | 0.02 | 1 | 50 | 0.02 |
| MSA | WV | Mason | 2016 | 0.039 | 0.131 | 21 | 22 | 50 | 0.44 | 22 | 50 | 0.44 |
| MSAR | WV | Mason | 2016 | 0.008 | 0.153 | 2 | 2 | 50 | 0.04 | 2 | 50 | 0.04 |
| MSB | WV | Mason | 2016 | 0.021 | 0.174 | 2 | 2 | 50 | 0.04 | 2 | 50 | 0.04 |
| MSBR | WV | Mason | 2016 | 0.022 | 0.146 | 35 | 40 | 50 | 0.80 | 40 | 50 | 0.8 |
| Ten Mile Creek | WV | Mason | 1999 | 0.497 | 0.484 | 10 | 10 | NA | NA | 10 | NA | NA |
| Thirteen Mile Creek | WV | Mason | 1999 | 5.577 | 0.331 | 3 | 3 | NA | NA | 3 | NA | NA |
| Three Mile Creek Left | WV | Mason | 1999-2000 | 2.723 | 0.154 | 14 | 14 | NA | NA | 14 | NA | NA |
| 13/3 | WV | Morgan | 2019 | 1.627 | 0.228 | 1 | 1 | 40 | 0.025 | 1 | 40 | 0.025 |
| Sleepy Creek WMA | WV | Morgan | 2019 | 2.003 | 0.6 | 5 | 5 | 40 | 0.125 | 5 | 40 | 0.125 |
| PKA | WV | Pendleton | 2017 | 0.011 | 0.373 | 2 | 2 | 50 | 0.04 | 2 | 50 | 0.04 |
| Fairfax Pond-Rehe WMA 1 | WV | Preston | 2018-2019 | 0.314 | 0.121 | 1 | 6 | 50 | 0.12 | 6 | 90 | 0.067 |
| Fairfax Pond-Rehe WMA 2 | WV | Preston | 2018-2019 | 0.23 | 0.168 | 2 | 4 | 50 | 0.08 | 4 | 90 | 0.044 |
| Fairfax Pond-Rehe WMA 3 | WV | Preston | 2018-2019 | 0.033 | 0.016 | 4 | 33 | 50 | 0.66 | 41 | 90 | 0.456 |
| Farm 3 | WV | Preston | 2018-2019 | 0.022 | 0.017 | 13 | 23 | 50 | 0.46 | 29 | 60 | 0.483 |
| Farm 4 | WV | Preston | 2018-2019 | 0.041 | 0.076 | 3 | 22 | 50 | 0.44 | 22 | 60 | 0.367 |
| Laurel Lake | WV | Preston | 2018-2019 | 0.153 | 0.331 | 5 | 21 | 50 | 0.42 | 21 | 60 | 0.35 |
| Pond at Zinn Chapel Rd 887 | WV | Preston | 2018-2019 | 0.009 | 0.193 | 2 | 2 | 50 | 0.04 | 2 | 60 | 0.033 |
| Pond Fairfax Pines Dr 22 | WV | Preston | 2018-2019 | 0.019 | 0.179 | 0 | 4 | 50 | 0.08 | 4 | 67 | 0.06 |
| Pond Kingwood Pike 4864 | WV | Preston | 2018-2019 | 0.022 | 0.288 | 0 | 2 | 50 | 0.04 | 2 | 60 | 0.033 |
| Pond Kingwood Pike 7032 | WV | Preston | 2018-2019 | 0.031 | 0.067 | 3 | 19 | 50 | 0.38 | 19 | 60 | 0.317 |
| PWA | WV | Preston | 2016 | 0.021 | 0.107 | 2 | 2 | 50 | 0.04 | 2 | 50 | 0.04 |
| PWB | WV | Preston | 2016 | 0.017 | 0.343 | 2 | 2 | 50 | 0.04 | 2 | 50 | 0.04 |
| PWBR | WV | Preston | 2016 | 0.023 | 0.043 | 2 | 2 | 50 | 0.04 | 2 | 50 | 0.04 |
| PWCR | WV | Preston | 2016 | 0.018 | 0.112 | 2 | 2 | 50 | 0.04 | 2 | 50 | 0.04 |
| Ruby Pond | WV | Preston | 2018-2019 | 0.047 | 0.143 | 3 | 8 | 50 | 0.16 | 10 | 90 | 0.111 |
| Upper Deckers Creek WMA 1 | WV | Preston | 2018-2019 | 0.081 | 0.071 | 2 | 11 | 50 | 0.22 | 14 | 60 | 0.233 |
| Upper Deckers Creek WMA 2 | WV | Preston | 2018-2019 | 0.078 | 0.091 | 2 | 12 | 50 | 0.24 | 12 | 60 | 0.2 |
| Wetland at Deckers Creek | WV | Preston | 2018-2019 | 0.063 | 0.103 | 1 | 16 | 50 | 0.32 | 21 | 60 | 0.35 |

*Site removed from relative abundance analysis to satisfy model assumptions.

Appendix 2. Model selection results for the influence of landscape integrity (landscape condition value [LCV]) on captures-per-unit-effort (CPUE) of painted turtles (*Chrysemys picta*) in West Virginia and red-eared sliders (*Trachemys scripta elegans*) in Texas. For CPUE, we used the full trapping dataset at each site (i.e., full CPUE analysis). We used Akaike’s Information Criterion corrected for small sample size (AIC*_c_*) to rank candidate models. For CPUE, we used the 2.5 km LCV and tested a linear and quadratic (Q) relationship. The size of traps (Trap Size) varied at West Virginia sites and was included as a candidate predictor for *C. picta*. The null model is shown as (.) and includes only the intercept. Akaike weights are represented as *w_i_*.

| Model | AIC*_c_* | ΔAIC*_c_* | w*_i_* |
| --- | --- | --- | --- |
| ***Chrysemys picta*** |  |  |  |
| *CPUE* |  |  |  |
| (.) | 1.35 | 0.00 | 0.62 |
| LCV_2.5_ | 3.39 | 2.03 | 0.22 |
| LCV_2.5_ + Trap Size | 5.73 | 4.37 | 0.07 |
| LCV_2.5_ (Q) | 5.76 | 4.40 | 0.07 |
| LCV_2.5_ (Q) + Trap Size | 8.20 | 6.84 | 0.02 |
|  |  |  |  |
|  |  |  |  |
| ***Trachemys scripta elegans*** |  |  |  |
| *CPUE* |  |  |  |
| LCV_2.5_ (Q) | -40.05 | 0.00 | 0.48 |
| LCV_2.5_ | -39.17 | 0.88 | 0.31 |
| (.) | -38.46 | 1.59 | 0.22 |


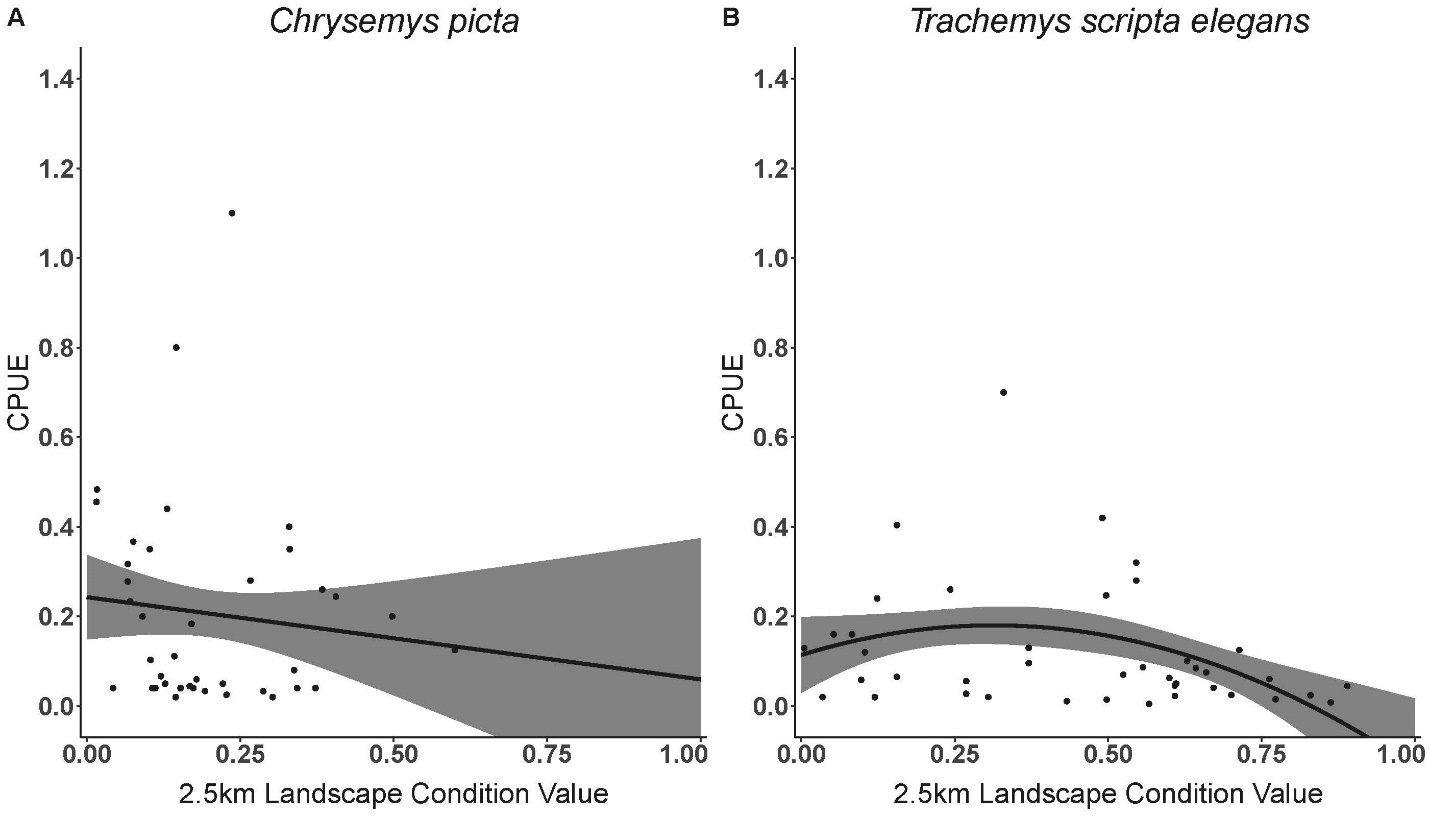


Appendix 3. Model-estimated relationship between mean 2.5 km landscape condition value (LCV) and captures-per-unit-effort (CPUE) for (A) 39 painted turtle (*Chrysemys picta*) wetlands located across 10 counties in West Virginia, and (B) 43 red-eared slider (*Trachemys scripta elegans*) wetlands located across 5 counties in Texas using the full trapping dataset at each site (i.e., full CPUE analysis). Wetlands where trap days could not be calculated were excluded from this analysis. We included wetland size as a random effect in analyses to account for the influence of size on CPUE. Black circles depict observed CPUE and gray bands depict 85% confidence intervals. Note the maximum LCV for *C. picta* sites was 0.6.
